# Rhizobia and non-rhizobial nodule bacteria with ACC deaminase increase both nodulation and stress resistance

**DOI:** 10.3389/fmicb.2025.1662592

**Published:** 2025-08-26

**Authors:** Elisa Gamalero, Bernard R. Glick

**Affiliations:** ^1^Università del Piemonte Orientale, Dipartimento di Scienze e Innovazione Tecnologica, Alessandria, Italy; ^2^Department of Biology, University of Waterloo, Waterloo, ON, Canada

**Keywords:** ACC deaminase, rhizobia, plant stress, heat, salinity, drought, toxic metals

## Abstract

Strains of *Rhizobia* that possess the enzyme 1-aminocyclopropane-1-carboxylate (ACC) deaminase facilitate the nodulation of cognate legume hosts. Some rhizobial strains that contain ACC deaminase also help plants to overcome some types of environmental stress including heat, salt, drought and the presence of heavy metals. In addition, non-rhizobial strains of bacteria isolated from legume nodules that contain ACC deaminase increase the extent of rhizobia nodulation and the resistance of the legume to environmental stresses. Here, the literature addressing the role of ACC deaminase in increasing legume nodulation and protecting plants against a range of environmental stresses is summarized and discussed.

## 1 Introduction

The large number of bacteria found in soil (~1 × 10^6^ to 1 × 10^9^ bacterial cells per gram of soil) includes a mixture of many strains of bacteria some of which are beneficial to plants (plant growth-promoting), some harmful to plants (pathogens) and others neutral to plant growth (Bulgarelli et al., 2013). The highest concentration of soil bacteria is generally found around the roots of plants since most plants exude into the soil a significant portion of the carbon that they fix through photosynthesis into small organic molecules, and the bacteria use this exuded carbon as a food source (Bais et al., 2006). Different plants attract unique cross-sections of the bacterial (and fungal) populations that are found in the soil, based on the unique composition of the root exudates produced by each plant (Glick and Gamalero, 2021). As a consequence, plants generally attract beneficial soil microorganisms to their rhizosphere and exclude potentially pathogenic microorganisms.

In the past 20–30 years scientists have endeavored to expand the use of beneficial plant growth-promoting bacteria (PGPB) as a means of developing environmentally friendly agricultural practice that does not depend upon the extensive use of potentially deleterious chemicals (Reed and Glick, 2023). For a start, this endeavor has involved the isolation and characterization of a large number of different PGPB with the goal of understanding the mechanisms that they utilize to facilitate plant growth (Glick, 2012). This approach requires a detailed knowledge of plant biochemistry and physiology including understanding the functioning of the phytohormones auxin, cytokinin, ethylene, jasmonic acid, gibberellin, salicylate, and abscisic acid (Ali et al., 2024). Thus, a key component of efficacious chemical-free agriculture includes developing a thorough understanding of the mechanisms used by PGPB to facilitate plant growth and development.

Central to the growth and development of plants, and especially their response to a range of environmental stresses is the plant hormone ethylene. The phytohormone ethylene and its immediate metabolic precursor molecule, 1-aminocyclopropane-1-carboxylate (ACC) are present in all higher plants, and in many primitive plants as well (Gamalero et al., 2023). Both ethylene and ACC play important roles in the development and growth of plants, especially during stressful conditions (Abeles et al., 1992). In this regard, the biosynthesis of ethylene in plants begins with the conversion of the amino acid L-methionine into the compound *S*-adenosylmethionine (SAM) by the enzyme SAM synthase (Fluhr et al., 2008; Pattyn et al., 2021). The compound SAM is then converted into ACC by the enzyme ACC synthase (Eun et al., 2019; Zarembinski and Theologis, 1994). Since plant cells often synthesize an excessive amount of ACC in comparison to the amount that they require for the production of ethylene, some of the ACC that is formed in plants is converted to inactive conjugated forms (Kende, 1989; Martin et al., 1995; Peiser and Yang, 1998; Yang, 1987).

Eventually, some of the ACC is converted into ethylene by the enzyme ACC oxidase (Yang and Hoffman, 1984). Ethylene is a key hormone in various aspects of plant growth and development and is especially important in a plant's response to both abiotic and biotic stresses (Abeles et al., 1992). Thus, “ethylene is involved in seed germination, tissue differentiation, formation of root and shoot primordia, root branching and elongation, lateral bud development, flowering initiation, anthocyanin synthesis, flower opening and senescence, fruit ripening and degreening, production of volatile organic compounds, …aroma formation in fruits, storage product hydrolysis, leaf senescence, leaf and fruit abscission, rhizobia nodule formation, mycorrhizae-plant interaction, and (importantly) the response of plants to various biotic and abiotic stress” (Abeles et al., 1992).

Until the more recent pioneering work of Kieber and his colleagues, it was believed that only ethylene, and not ACC, could act as a plant growth regulator (Xu et al., 2008; Yoon and Kieber, 2013; Polko and Kieber, 2019). However, it is now clear that ACC can also act as a signaling hormone, albeit in a limited number of instances; this is in comparison to the multiplicity of roles played in nature by ethylene as a signaling molecule (Binder, 2020). Thus, it has been suggested that ACC may have been a major signaling molecule in primitive plants prior to the evolution of ethylene and ethylene signaling (Gamalero et al., 2023). In this regard, ACC currently appears to retain only a small vestige of that purported early hormonal activity. Therefore, the major focus of this article is the role that ethylene and its modulation of plant growth and development plays in rhizobia-plant interaction and subsequent plant development and growth. In this regard, our emphasis, but not exclusive focus, will be on manuscripts published within the past 5–10 years.

## 2 ACC deaminase

Plants are highly dependent upon beneficial soil microorganisms, i.e., PGPB and mycorrhizal fungi, for their ability to grow and develop including during periods of environmental stress, both biotic and abiotic (Ali and Glick, 2021). Various PGPB use a range of different mechanisms to facilitate plant growth and development including synthesizing auxin, cytokinin and gibberellin, fixing atmospheric nitrogen, solubilizing iron, phosphorus and potassium (and other nutrients) from the soil, and decreasing or preventing the inhibitory effects of plant pathogens on plants including the negative effects from deleterious fungi, bacteria, nematodes, and insects (Glick, 2012; Singh et al., 2018).

**Figure 1:**
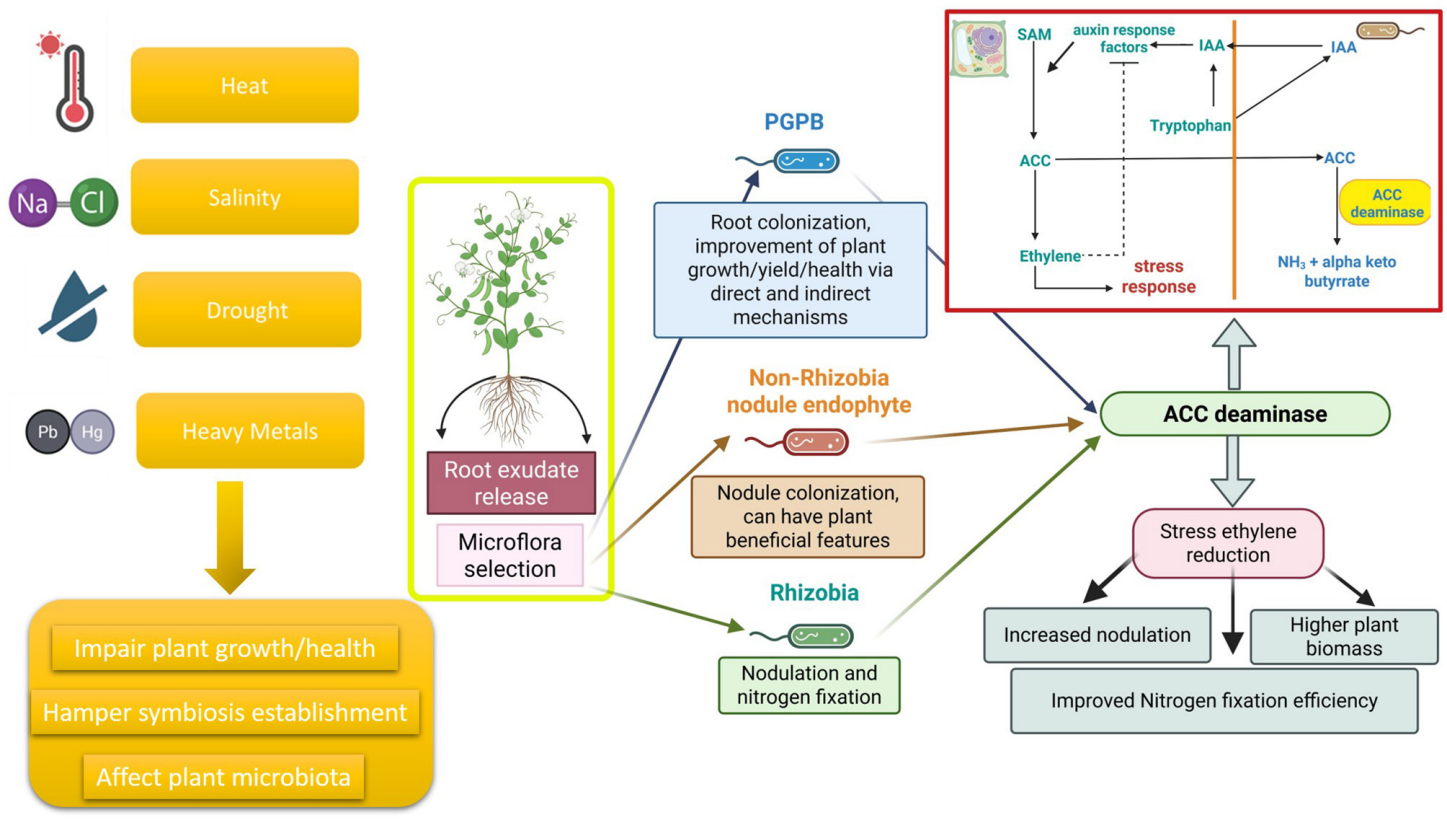
Plant roots release root exudates that exhibit a chemoattractive effect toward a diverse microflora. that become unique of the plant according to the plant species, the phenological stage and its health status. Various abiotic and biotic stress factors, such as high temperature, salinity, drought and heavy metal pollution, could impair the establishment of the legume-rhizobia symbiosis, as well as plant growth and health and affect the composition of the microbiota. Rhizobia, Non-Rhizobial Endophytes (NREs) or PGPB expressing ACC deaminase can alleviate plant stress induced by all these factors by lowering the levels of stress ethylene according to a metabolic pathway well described by Glick et al. (2007) and reported in the red box. It has been widely demonstrated that mutants lacking ACC deaminase are unable to support plants facing such stressful conditions through the reduction of ethylene level. Despite the potentiality of the system and the increasing importance of these stressors, especially under climate change, at our knowledge, no scientific papers describing the effect of rhizobia expressed ACC deaminase on legume plants facing cold stress, flooding, organic pollution, pest attack and nematode colonization have been published. This figure was created with BioRender.com.

Arguably, the key metabolic trait that enables some PGPB to efficiently promote plant growth is the presence of the enzyme ACC deaminase (commonly present in many soil bacteria and some fungi) (Glick, 2012, 2015; Gamalero and Glick, 2015; Nascimento et al., 2021; Shahid et al., 2023). ACC deaminase cleaves the compound ACC, to ammonia and α-ketobutyrate, so that it can no longer be converted to ethylene. This prevents ethylene from accumulating within plants and thereby inhibiting plant growth. A model that explains the role of bacterial ACC deaminase in promoting plant growth that was previously proposed (Glick et al., 1998) indicated that (1) ACC deaminase-producing PGPB typically bind to the roots of plants. (2) In response to tryptophan and other small molecules from the plant root exudates, the PGPB synthesize and secrete the phytohormone indole-3-acetic acid (IAA), some of which is taken up by the plant. (3) This IAA, together with endogenous plant-synthesized IAA can stimulate plant cell proliferation and/or plant cell elongation, and it induces the transcription of the plant enzyme ACC synthase catalyzing the synthesis of additional ACC within the plant. Thus, IAA stimulates ethylene synthesis (Yu and Yang, 1979) and concurrently loosens plant cell walls (Kutschera and Briggs, 1987), facilitating plant cell elongation and increasing the amount of root exudation. (4) Some of the plant ACC is exuded (Penrose and Glick, 2001) and taken up by the ACC deaminase-containing PGPB and then cleaved by ACC deaminase. ACC cleavage by bacterial ACC deaminase means that the bacterium is acting as a sink for excess ACC. (5) The amount of ethylene that might have formed in the plant is reduced as is the ethylene inhibition of plant growth following a wide range of environmental stresses (Figure 1).

Consequently, plants grown together with ACC deaminase-containing PGPB typically have longer roots and shoots and are more resistant to growth inhibition by a variety of ethylene-inducing environmental stresses (e.g., Timmusk et al., 2011). The presence of ACC deaminase was first reported in rhizobia more than 20 years ago by Ma et al. (2003a). In that study, the presence of ACC deaminase was ascertained by Southern hybridization, Western blots and ACC deaminase enzyme assays, using the free-living bacterium *Pseudomonas* sp. UW4 (Duan et al., 2013) as a positive control. Interestingly, a few rhizobia strains in that study were positive for ACC deaminase by Southern hybridization but negative for ACC deaminase according to the results of Western blots and enzyme assays. The presence of an ACC deaminase gene in those rhizobial strains was eventually confirmed when the strains' genomes were sequenced.

The infection of legume plant roots by plant-specific rhizobia strains causes plants to locally produce low levels of ethylene which inhibits subsequent legume nodulation (Hirsch and Fang, 1994; Guinel and Geil, 2002). However, a few strains of rhizobia naturally produce either rhizobitoxine [2-amino-4-(2-amino-3-hydropropoxy)-transbut-3-enoic acid], a chemical inhibitor of the enzyme ACC synthase, or ACC deaminase both of which allows these bacterial strains to lower the ethylene levels in the plant and increase subsequent nodulation (and biomass formation) (Nukui et al., 2000; Yuhashi et al., 2000; Ma et al., 2003b). In addition, surveying different rhizobia strains for ACC deaminase activity revealed that many commercial strains, but only a very few field strains, had this activity (Duan et al., 2009). This suggests that the direct screening of field rhizobial isolates for ACC deaminase activity may be one means of rapidly selecting more efficient strains of rhizobia. Alternatively, the addition of an ACC deaminase-containing free-living bacterium together with a nodule-forming rhizobia strain not only increases the extent of legume nodulation and decreases the subsequent rate of nodule senescence, but also provides the plant with protection from a range of environmental stresses (Nascimento et al., 2012; Tavares et al., 2018; Nascimento et al., 2018, 2019).

Interestingly, when beneficial free-living soil bacteria (i.e., PGPB) contain the enzyme ACC deaminase, they typically demonstrate a 10-to 30-fold higher level of ACC deaminase activity than do nodule-forming rhizobia (Glick et al., 2007; Shahid et al., 2023) whose activity is not always readily detected in enzyme assays. This likely reflects the fact that the amount of ACC deaminase that is synthesized in the nodule is only a small fraction of the amount of enzyme that is produced by free-living bacteria. ACC deaminase produced by nodule-forming rhizobia facilitates nodulation by decreasing the localized level of ethylene by breaking down the local level of ACC. Thus, while both free-living bacteria and nodule-forming rhizobia can both facilitate nodulation, the free-living bacteria generally lower ethylene levels throughout the plant while the ACC deaminase-containing nodule-forming rhizobia does not affect overall plant ethylene levels. It is thought, that since the enzyme synthesized in both cases is essentially identical (Ali and Glick, 2021), this difference in ACC deaminase activity is a direct result of differences in the transcriptional regulation between free-living bacteria and nodule-forming rhizobia (Shahid et al., 2023). In many free-living bacteria ACC deaminase expression is transcriptionally quite complicated. It is controlled by an LRP protein (that is synthesized by the *acdR* gene) which binds to a DNA sequence known as an LRP box that overlaps the *acdR* promoter sequence preventing further transcription of this gene. Alternatively, the LRP protein can bind to a complex of ACC and the AcdB protein with this tripartite complex binding to either an FNR box (on the bacterial DNA) in the absence of oxygen, or to a CRP box (also on the bacterial DNA) in the presence of oxygen, either of which can activate the transcription of *acdS* the structural gene for ACC deaminase (Glick et al., 2007). On the other hand, in some strains of rhizobia the ACC deaminase structural gene (*acdS*) is under the transcriptional control of a *nifA* promoter [which also controls the transcription of nitrogen fixation (*nif* ) genes] (Ma et al., 2002; Nukui et al., 2006). Here, the *nifA* promoter consists of *nifA1* and *nifA2* promoters positioned upstream of the *acdS* gene with a σ54 RNA polymerase recognition site. It has been suggested that the *nifA* promoter interacts with a σ54 RNA polymerase thereby promoting *acdS* transcription. This mode of regulation may benefit the nodules that contain these rhizobia strains in preventing their premature senescence that might otherwise be caused by excessive ethylene levels. The above comments notwithstanding, some strains of rhizobia have more than a single ACC deaminase gene and a few of these rhizobia strains have a level of ACC deaminase activity that is normally associated with free-living bacteria.

## 3 Rhizobia, non-rhizobia nodule endophytes and plant stress

While the legume-rhizobia symbiosis is crucial for legume productivity, especially in nitrogen-deficient soils, environmental stresses such as salinity, drought, heat, and heavy metal contamination can severely impair this symbiosis, reducing nodulation efficiency, nitrogen fixation, and overall plant growth. One key mechanism by which some rhizobia can alleviate stress effects is the production of ACC deaminase, and the reduction of plant ethylene levels. Frequently, root nodules are colonized not only by rhizobia, but also by bacteria collectively known as Non-Rhizobia Endophytic (NRE), including genera as *Pseudomonas, Enterobacter, Bacillus, Pantoea, Niastella, Shewanella, Ohtaekwangia, and Rhizobacter* (Brown et al., 2020; Hassen et al., 2025), that are recognized as opportunistic or transient root nodule colonizers (Novello et al., 2022; Dhole and Shelat, 2022; Debnath et al., 2023). Though originally considered opportunistic colonizers, many NREs have been shown to possess plant growth-promoting traits, including ACC deaminase activity (Singh et al., 2022). These bacteria can act synergistically with rhizobia to enhance plant tolerance to abiotic stress, improve nodulation, and stimulate plant growth (Paço et al., 2020). Based on the idea that inoculation or co-inoculation of legume plants with rhizobia or/and selected NREs able to produce ACC deaminase is a promising tool for sustainable agriculture especially in stress-prone regions, in the next sections we will detail the most recent works dealing with rhizobia, non-rhizobia nodule endophytes and plant stress such as high temperature, salinity, drought and toxic metals.

### 3.1 Heat

According to the Food and Agriculture Organization (FAO) of the United Nations (https://library.wmo.int/records/item/66214-state-of-the-global-climate-2022#.ZEZiSXZBw2z), once the current year has ended, the past 8 years are on track to be the eight warmest years on record, and this warming is significantly impacting global food production, leading to crop failure and high economic costs. In this regard, it is surprising to observe that the literature about the involvement of ACC deaminase synthesized by rhizobia or non-Rhizobia endophytes in the tolerance to high temperature is limited to one paper. In a recent study, Ben Gaied et al. (2024) assessed the effect of three non-rhizobial endophytes isolated from legumes identified as *Phyllobacterium salinisoli* (PH), *Starkeya* sp. (ST), and *Pseudomonas turukhanskensis* (PS) on the growth of *Pisum sativum* (pea plants) cultivated under heat stress and on the symbiosis establishment of *Rhizobium leguminosarum* 128C53 (wild-type) and its ACC deaminase-deficient mutant on the roots of these plants. In these experiments, pea plants were inoculated with different combinations of the bacterial strains (i.e., ST + PS, ST + PH, PS + PH, and ST + PS + PH) plus *R. leguminosarum* 128C53 or its *acdS* minus mutant and exposed to heat stress for 15 days. In detail, for 2 weeks plants were grown in a growth chamber under optimal conditions (photoperiod of 16 h/24 °C for the day cycle and 8 h/18 °C for the night). Then, a heat stress was imposed with cycles of 30 to 35 °C for 16 h (day cycle), with intervals of 30 °C for 6 h, 32 °C for h, and 35 °C for 4 h, followed by 20 °C for 8 h during the night. The results obtained showed a low root biomass and a low level of plant nodulation efficiency in plants inoculated only with the ACC deaminase minus mutant suggesting a negative impact on both plant growth and nodule formation in the absence of ACC deaminase under heat stress conditions. Unfortunately, no growth promotion effect was observed in pea plants inoculated with the endophyte consortium and the rhizobia able to produce ACC deaminase. However, treating plants with the ACC deaminase minus mutant and the endophyte consortium led to synergistic interactions on plant performance suggesting that this mixed inoculum overcame the absence of rhizobial ACC deaminase and at the same time improved pea tolerance to heat stress possibly through other mechanisms such as IAA or improvement of plant nutrition and water uptake. No positive effects were observed when the endophyte consortium was combined with the wild-type strain of *R. leguminosarum*. Overall, this interesting work highlights the potential of non-rhizobial endophytes to improve symbiotic performance of rhizobial strains lacking genetic mechanisms involved in stress relief on their legume host.

### 3.2 Salt

In 2024, nearly 1.4 billion hectares of land (corresponding to about 10% of the global land area) are negatively affected by salinity (https://www.fao.org/newsroom/detail/fao-launches-first-major-global-assessment-of-salt-affected-soils-in-50-years/en). The main adverse effect of salt stress on plants is related to osmotic toxicities and to the uptake of ions in toxic concentrations (Wekesa et al., 2022) leading to a limitation of water and nutrient uptake by the root system. Legume plants show high sensitivity to soil salinity, and this happens especially in Mediterranean countries, where salt stress is considered as one of the most important abiotic factors hampering legume yield (Nadeem et al., 2019) and grain quality (Jha et al., 2024). Moreover, several researchers reported that soil salinity reduced nodulation of legumes as well as nitrogen fixation efficiency (Abd-Alla, 1992; Sharaf et al., 2019; Kirova and Kocheva, 2021). Given the fact that the level of ACC deaminase activity in rhizobia, if present, is considerably less than in free-living PGPB (Glick and Stearns, 2011) plant inoculation with a bacterial consortium including both rhizobia and an ACC deaminase-containing PGPB is a good means of improving legume growth and health in stressful conditions. In this context, salt tolerant *Rhizobia* strains synthesizing ACC deaminase, alone or in a consortium with other plant beneficial microorganisms are ideal candidates for legume cultivation on saline soil.

The effect of a mixed inoculum including the salt tolerant *Rhizobium* sp. LSMR-32 and *Enterococcus mundtii* LSMRS-3, both producing ACC deaminase, although at a different extent, was assessed on the growth and yield of spring mungbean (Kumawat et al., 2021). A 3-year field experiment was carried out in a saline soil in the Punjab region of India by growing plants inoculated with each of the two bacterial strains alone or the two strains in combination. Plants treated with the consortium and grown in the saline soil showed a higher level of seed germination level, plant height and biomass, chlorophyll content and macro/micro-nutrient uptake, compared to uninoculated plants. Other parameters regarding the rhizobia-legume symbiosis such as nodulation degree, nodule biomass and leghemoglobin and grain yield were also significantly higher in co-inoculated plants compared to plants inoculated only with the *Rhizobium* sp. LSMR-32 strain. Moreover, higher amounts of proline and several anti-oxidative enzymes were observed in plants treated with the bacterial consortium.

Similarly, the effects of a consortium including *Sinorhizobium meliloti* GL1 and *Enterobacter ludwigii* MJM-11 on the productivity, nodulation efficiency and quality of alfalfa cultivated in a saline-alkali soil (pH 9.07; total Na 52.31 mg/Kg) was evaluated by Gao et al. (2023). Both of these strains produced ACC deaminase, although to a different extent (with strain MJM-11 the observed enzymatic activity was four times higher than with strain GL1). However, co-inoculation in the growth medium required by the test, lead to a significantly higher level of enzyme activity compared to single inoculation. Plant inoculation with the two bacterial strains in pot trials showed that the bacterial consortium increased plant growth and favored the establishment of the symbiosis between the rhizobial strain and alfalfa, then subsequently increasing the nitrogenase activity inside the root nodule. In fact, by using CLSM (Confocal Scanning Laser Microscope) it was observed that the occupancy rate of rhizobia in the plant nodules inoculated with the consortium was 18.45% higher than in the nodules of plants inoculated only with strain GL1. The impact of the co-inoculation with the two bacterial strains on the yield and quality of alfalfa was assessed by growing plants in a saline-alkali soil, in an open field experiment. While the total yield, the crude protein level and the phosphorus content of alfalfa inoculated with each of the bacterial strains and co-inoculated with both of them increased by 26.12%, 24.32%, and 20.61%, respectively, the neutral and acid detergent fiber content was reduced by 10.83% and 11.87%, respectively, compared to uninoculated control plants. Overall, the results described in this work indicate a synergy between rhizobia and other PGPB, in legumes cultivated in saline-alkaline soils.

Synergistic effects have been observed in soybean by testing the dual application of two *Bradyrhizobium* strains (*B. diazoefficiens* USDA110 and *B. ottawaense* SG09) and plant growth-promoting *Pseudomonas* spp. OFT2 and OFT5, able to synthesize ACC deaminase, under both normal and saline conditions (Win et al., 2023). Soybean seedlings were inoculated with each of the rhizobia strains and with the rhizobia strains together with the pseudomonads in pots irrigated with a nutrient solution containing 0 or 60 mM NaCl. When strain *Bradyrhizobium* USDA110 was inoculated together with the two pseudomonads, nitrogen-fixation was enhanced by 11% and 56%, respectively. An improved performance was also obtained by inoculating the pseudomonads with *Bradyrhizobium* strain SG09; in this case, the level of nitrogen fixation increased by 76% and 81%, respectively. Plants exposed to salinity suffered from the imposed stress showing limited growth, nodulation and nitrogen fixation. While no beneficial effect was observed on plant growth and health by the treatment with USDA110 and OFT5, the consortium of each pseudomonad together with strain SG09 lead to a significant level of stress relief highlighted by a reduction of ethylene synthesis resulting from the presence of salinity and to the improvement of nutrient uptake, nodulation, and N_2_-fixation. More in detail, the nodule number and dry biomass in plants treated with *Pseudomonas* spp. OFT2 and OFT5 combined with *Bradyrhizobium* strain SG09 and exposed to salt were twice the level observed with soybean plants inoculated with *B. ottawaense* SG09 alone. The results reported in this paper demonstrated that, under optimal growth condition (i.e., no stress), the combined inoculation of each rhizobial strain with each pseudomonad improves both nodulation and N_2_-fixation in soybean plants. However, when plants are subjected to salinity stress, only the combination of strain *B. ottawaense* SG09 with either *Pseudomonas* sp. OFT2 or *Pseudomonas* sp. OFT5 induces positive effects on plant development and nitrogen fixation efficiency (Win et al., 2023).

Examining the role of rhizobia able to synthesize ACC deaminase, in the alleviation of salinity stress, Alinia et al. (2022) assessed the capability of *Rhizobium leguminosarum* bv. *phaseoli* to overcome the stress imposed by different salinity levels (2.6, and 5.1 g L^−1^ NaCl) in common bean plants treated with 100 μM melatonin. While salt stress imposed a significant reduction of plant growth and yield coupled with a decrease of nitrogen fixation efficiency, plant treatment with melatonin and the rhizobium strain increased both plant growth and photosynthesis as well as nitrogen fixation efficiency. Moreover, plants inoculated with *R. leguminosarum* and treated with melatonin and then exposed to salt stress showed higher values of growth-related biochemical parameters such as nitrogen and protein content in shoots and roots, compared to untreated plants. These results demonstrated that under salinity stress the plants treated with both melatonin and rhizobia perform better than plants treated with either the hormone or the bacterial strain alone.

In their work, Oviya et al. (2023) assessed the effects of NRE including the species *Pantoea dispersa* YBB19B and *Bacillus tequilensis* NBB13 and rhizobia strains (*Rhizobium phaseoli* S18 and *Rhizobium pusense* S6R2) on the growth of groundnut exposed to salt stress. All of these bacterial strains were able to express plant beneficial activity, including ACC deaminase, both in the absence and presence of 3% NaCl, with the strain *Rhizobium phaseoli* S18 (surprisingly) being the best producer of this enzyme. In these experiments, the plants received nine different treatments (including the uninoculated control, each of the bacterial strains, and the combination of Rhizobia with the NRE strains) and were cultivated in a saline soil in both pots and under open field conditions with an EC of 4.32 dS m^−1^and pH 8.4. In the pot culture experiment, the combined inoculum *R. pusense* S6R2 and *P. dispersa* YBB19B increased both shoot and root lengths as well as the pod and the nodule numbers. However, the dry biomass of the groundnut plants was highest in plants treated with *R. phaseoli* S18 and *P. dispersa* YBB19B. Accordingly, the results of the experiment performed in field conditions highlighted the shoot length enhancement by *R. pusense* S6R2 + *P. dispersa* YBB19B and the increased root length in plants inoculated with strains *R. phaseoli* S18 and *P. dispersa* YBB19B. Moreover, the consortium of *R. pusense* S6R2 and *P. dispersa* YBB19B increased the nodule number per plant and the pod number per plant compared to the uninoculated control. The highest level of productivity was measured in plants receiving the *R. phaseoli* S18 + *P. dispersa* YBB19B consortium. Although the clear involvement of ACC deaminase in salt stress relief was not demonstrated in these experiments, the results discussed in this paper suggested that salt stress relief was efficiently induced by the consortium of *R. pusense* S6R2 and *P. dispersa* YBB19B.

This brief analysis of the limited literature dealing with ACC deaminase-containing rhizobia demonstrates that the application of salt-tolerant *Rhizobium* strains able to synthesize ACC deaminase, either alone or in combination with either other plant growth-promoting bacteria, biostimulants, or non-rhizobial endophytes represents a promising strategy to mitigate the detrimental effects of salinity on legume growth and productivity. In this regard, the existing experimental evidence highlights the improvements observed in plant vegetative growth, nodulation, nitrogen fixation efficiency, and overall plant yield and quality of legume crops in the presence of ACC deaminase. Therefore, microbial consortia combining salt-tolerant rhizobia and ACC deaminase-producing PGPB emerge as effective tools for sustainable legume cultivation in saline environments, especially in vulnerable regions such as the Mediterranean basin.

### 3.3 Drought

Drought is widely recognized as the most significant factor contributing to agricultural yield loss (https://openknowledge.fao.org/server/api/core/bitstreams/069ceb86-59b2-4b6e-90e0-b7bd26a58c76/content). In fact, it has been estimated that ~34% of crop and livestock production loss in the world's least developed countries is due to water scarcity, accounting for an annual loss of US$ 37 billion. As one means of addressing this problem, the utilization of microorganisms as biofertilizers is gaining more and more importance. Although legumes vary widely in their sensitivity to drought, inoculating them with ACC deaminase producing bacteria under limited water conditions can help to reduce the negative impacts of drought on plant yield, nodule development, and nitrogen fixation (López et al., 2023).

A clear example of overcoming some of the inhibitory effects of drought on plant growth and development is provided by a study assessing the impact of two bacterial isolates belonging to the genera *Rhizobium* and *Pseudomonas*. These bacteria were used either alone or in combination with P-enriched compost, on the productivity of chickpea grown under drought stress conditions in an open field in Bahawalpur, Pakistan (Ahamd et al., 2017). While both individual and combined treatments improved nodule number and plant biomass, the co-application of the two bacterial strains with P-enriched compost proved to be the most effective treatment in boosting chickpea growth, the number of pods and grains, the number of nodules per plant and the average nodule dry weight compared to untreated plants. Together with these results on plant growth and productivity, the combined inoculum of bacterial isolates able to synthesize ACC deaminase and P-enriched compost also enhanced the nitrogen and phosphorus contents of plants. Overall, the information described in this manuscript led to the conclusion that inoculating legume plants subjected to drought stress with ACC deaminase producing bacteria could facilitate chickpea development, while improving its productivity (Ahamd et al., 2017).

In agreement with these results, Belimov et al. (2019) described the effects of *Rhizobium leguminosarum* by. *viciae* 1066S, able to produce ACC deaminase and its mutant RIM1 unable to synthesize the ACC deaminase with both the wild-type and the mutant able to tolerate 0.5 μM cadmium (CdCl_2_), on pea plant lines sensitive (SGE) and tolerant [SGECd(t)] to both this heavy metal and to drought stress. The two lines of pea plants were inoculated with the rhizobia strain 1066S or its ACC deaminase minus mutant and subjected or not to a water limited condition and cadmium contamination. Inoculation with rhizobia strain 1066S significantly improved shoot biomass and nutrient uptake, as well as the nodulation rate and nitrogen fixation efficiency, compared to uninoculated controls subjected to drought or cadmium contamination. The nodule number per plant in both the cadmium sensitive and tolerant genotypes grown in water deficit and inoculated with the ACC deaminase-containing rhizobia strain 1066S was 4–5 times higher than uninoculated controls, and about 2 times bigger than plants inoculated with the ACC deaminase minus mutant. Especially in the pea plant line tolerant to Cd the inoculation with rhizobia led to increased shoot Cd content. When both drought and cadmium stress were present, the efficacy of the inoculum was reduced, and optimal plant growth could not be restored in the Cd sensitive pea genotype. The great merit of this research study is the demonstration, for the first time, that the enzyme ACC deaminase expressed by rhizobia is fundamental for achieving successful nodulation in pea plants exposed to stressful conditions such as water scarcity and heavy metals.

The effect of a microbial consortium including *Pseudomonas putida* and *Bradyrhizobium japonicum*, both able to synthesize ACC deaminase, and an arbuscular mycorrhizal fungus (*Glomus intraradices*, now *Rhizophagus irregularis*) on fenugreek (an herb with seeds used in Indian and Middle Eastern cooking) growth under drought stress was evaluated in a study by Irankhah et al. (2021). These authors focused their attention on the content of diosgenin [a bioactive molecule with antidiabetic, hypocholesterolemic, anti-inflammatory (Khorshidian et al., 2016), and anti-cancer activity (Lohvina et al., 2012) in fenugreek plants]. The amount of diosgenin was found to be higher in leaves under non-stressed conditions compared to leaves of plants cultivated under drought stress, with the highest concentration observed in plants grown in non-drought conditions and inoculated with both rhizobia and the AM fungus. Although the highest diosgenin levels were observed in inoculated plants under optimal moisture, the specific involvement of ACC deaminase was not clearly established, limiting the conclusions of the study.

In one study, 98 drought tolerant bacterial strains were first isolated from mungbean root nodules. Among them, 24 isolates tolerated 40% polyethylene glycol (PEG)-6000, a polymer simulating drought stress, and 21 isolates survived at a temperature of 45 °C, however, only 8 strains survived at the combined stresses (45 °C and 40% PEG-6000). Twenty-six of the bacterial isolates that were able to tolerate drought or temperature stresses were further characterized for their plant beneficial activities (IAA synthesis, N_2_ fixation, phosphate solubilization, and ACC deaminase production). Surprisingly, 23 of the 26 tested bacterial strains harbored all of abovementioned plant beneficial activities with ACC deaminase activity being the least frequently recorded activity. Four bacterial isolates *Rhizobium* sp. MuJs52b, *Rhizobium* sp. MuJs53b, *Rhizobium* sp. MuJs72a, and *Pseudomonas indica* MuBk32b, all showing plant beneficial activities were used to inoculate mungbean seeds in order to assess their effect on the growth of plants cultivated in pots and exposed to drought stress. Mungbean plants grown under low water availability and inoculated with these microorganisms showed the highest nodule and shoot dry weight compared to the other strains. However, notwithstanding the extensive data that was collected, the specific role of ACC deaminase in these improvements was not conclusively demonstrated (Mondal and Gera, 2024).

Naively, it was previously thought that rhizobia were the sole bacteria found in root nodules. However, to date several papers reported that Non-Rhizobia Endophytic Bacteria (NRE) can transiently colonize root nodules by entering through the infection threads, which are induced by rhizobia (Ibáñez et al., 2017; Novello et al., 2022). In their study, Ramakrishnan et al. (2024) used the bacterial strain *Rhizobium pusense* S6R2 alone or associated with the NRE isolates *Enterobacter cloacae* S23 and *Bacillus tequilensis* NBB13 as inoculants of groundnut (*Arachis hypogaea)*. These bacterial strains were selected based on their drought resistance measured on PEG 6000 and the expression of plant beneficial traits (IAA, exopolysaccharide, capability to form biofilm, ACC deaminase synthesis, P and Zn solubilization) under drought stress conditions. Following testing, the consortium of *R. pusense* and *E. cloacae* significantly enhanced groundnut development under water scarcity; this included increasing the number of nodules, photosynthetic pigment levels and antioxidant enzymes, compared to all the other treatments.

The ability of ACC deaminase expressing rhizobia, alone or in consortia with other bacterial species, has been consistently demonstrated across various studies to enhance nodulation, nitrogen fixation, and overall plant productivity under water-limited conditions. While the effectiveness of these microbial inoculants has been clearly observed in some studies including chickpea, pea, mungbean, and groundnut, the precise contribution of ACC deaminase activity has not always been definitively proven compared to other plant growth-promoting traits. Nevertheless, the overall evidence clearly points to the role of ACC deaminase in supporting symbiotic efficiency and stress tolerance in legumes, especially when applied as part of selected microbial consortia combining multiple synergistic PGP traits.

### 3.4 Metal contaminants

Toxic metals can severely impair plant growth and microbial activity in soils (Campillo-Cora et al., 2025). For the rhizobia/legume symbiosis the main effects of toxic metals are the inhibition of rhizobial survival, reduction of root hair formation, and interference with the nodulation and nitrogen fixation processes leading to an inhibition of plant growth and development (Pal, 1996; Broos et al., 2005; Sheirdil et al., 2012). However, it is also true that the legume-rhizobia symbiosis has stirred the attention of scientists working on remediation of toxic metals polluted soils. In fact, growing legume plants inoculated with metal resistant bacterial strains, especially rhizobia, in soils polluted by toxic metals is considered a tool to enhance phytoremediation efficiencies (Fagorzi et al., 2018). In particular, the enzyme ACC deaminase, synthesized by rhizobia can mitigate some of the harmful effects of heavy metals and support the symbiosis process under these stressful conditions. Despite the importance of these interactions, the scientific literature exploring the combined effects of heavy metals, legume–rhizobia symbiosis, and ACC deaminase activity remains scarce and fragmented, highlighting the need for more targeted studies. The main results obtained to date in this field are summarized below.

As mentioned previously, the amount of ACC deaminase produced by rhizobia is typically much lower than the amount of this enzyme synthesized by many free-living plant growth-promoting bacteria. Thus, the ACC deaminase level typically found in rhizobial strains is often insufficient to support plant growth under stressful conditions (Glick et al., 2007). In one study, Kong et al. (2015) evaluated the impact of a strain of *Sinorhizobium meliloti* genetically engineered to overproduce ACC deaminase on its symbiotic efficiency in *Medicago lupulina* (commonly black medic) grown under either moderate (200 mg/Kg) or severe (400 mg/Kg) levels of inhibitory copper. *M. lupulina* seedlings were grown in the presence or absence of copper and were inoculated with either the wild-type strain of *S. meliloti* CCNWSX0020 or with its ACC deaminase overproducing transformant and harvested after 40 days. The plants were then evaluated for plant biomass, root length, nodule number, nodule fresh weight, nitrogenase activity and plant nitrogen content. As expected, severe copper stress reduced the symbiotic efficiency of the bacterium and the plant. However, inoculation with the ACC deaminase overexpressing strain induced increased plant development, as measured by increased dry weight, and a reduced amount of root ethylene as well as a higher accumulation of copper in the root system compared to plants treated with the wild-type rhizobial strain. In detail, the shoot biomass in plants inoculated with the ACC deaminase overexpressing strain was increased by 31.6% under moderate stress and by 54.4% in severe stress compared with the plants treated with the wild-type strain of *S. meliloti*. Similar effects were observed on root weight (+34.6 and 39.4% compared to plants inoculated with the wild-type). The inhibitory level of copper induced an increase in ethylene production in both plant shoots and roots inoculated with the wild-type rhizobial strain. The ethylene level in both the aerial parts and roots of plants inoculated with the ACC deaminase overexpressing strain was significantly reduced compared to uninoculated plants.

Two different genotypes of pea seeds, one for plants sensitive to cadmium (SGE) and the other tolerant to the metal [SGECd(t)] were inoculated with a bacterial consortium including two ACC deaminase-containing bacteria, *Variovorax paradoxus* 5C-2 and *Rhizobium leguminosarum* bv. *viciae* RCAM1066, and the arbuscular mycorrhizal fungus *Glomus* sp. 1Fo. The growth of the cadmium tolerant pea plants, exposed to 15 mg/Kg of cadmium and treated or not with the bacterial consortium was compared with the development of wild-type pea and the line VIR263 of Indian mustard (*Brassica juncea* L. Czern.) able to accumulate cadmium in its tissues. Cadmium contamination reduced the growth of inoculated and uninoculated pea SGE plants, while no adverse effects on plants were observed in the SGECd^t^ mutant, except for reduced development of root systems (−15%) in inoculated plants. However, the biomass of pea plants belonging to the two genotypes and inoculated with the microbial consortium was twice the level of uninoculated controls irrespective of the metal contamination. Cadmium affected *B. juncea* plant growth to a lesser extent than it affected pea plants. The cadmium tolerant plants accumulated a higher concentration of cadmium compared to the cadmium sensitive plants. Regarding the effect of cadmium on the plant physiology, the metal reduced the number of nodules as well as the level of nitrogen fixation in the cadmium sensitive plants by 5.6 and 10.8 times, and by 2.1 and 2.8 times in the cadmium tolerant line. Moreover, the occurrence of mycorrhizal structures on plant roots was found to be decreased only in cadmium sensitive line. Finally, the microbial consortium improved nutrient uptake and accumulation in plants exposed to cadmium contamination, especially in the cadmium tolerant line (Belimov et al., 2020).

Abandoned mine tailings are of particular environmental concern as they represent a long-term ongoing source of heavy metals that can leach into surrounding soils and water bodies posing risks to ecosystems and agricultural productivity. With this in mind, Alami et al. (2025) proposed a phytoremediation plan based on phytostabilization performed by native legume species and their associated microbiota. This strategy includes the isolation and characterization of 40 bacterial strains from nodules of *Astragalus armatus* (a perennial shrub known as thorny milkvetch and native to parts of north Africa) grown in tailings of an abandoned mining site. Among the isolated bacterial strains, 26 strains were identified as rhizobia with six strains identified as *Mesorhizobium* sp. The six *Mesorhizobium* strains were found to have plant beneficial traits, including ACC deaminase, and to tolerate high concentrations of lead and zinc. Plant inoculation with these isolates favored plant growth and increased the content of chlorophyll in *A. armatus* grown in metal-rich mine tailings.

Therefore, harnessing ACC deaminase activity—whether via naturally occurring strains or genetically enhanced rhizobia—can substantially ameliorate metal-induced stress and support plant growth and symbiotic performance under conditions of moderate to severe contamination. Moreover, microbial consortia, including ACC deaminase–producing bacteria and mycorrhizal fungi, demonstrate synergistic benefits in both metal-sensitive and -tolerant genotypes, improving biomass, nutrient uptake, and nodulation even in metal-contaminated soils (Belimov et al., 2020). The promising results obtained in controlled conditions are further reinforced by phytoremediation strategy application in abandoned mine tailings, where native legume species inoculated with metal-tolerant rhizobia exhibiting ACC deaminase activity demonstrated enhanced establishment and chlorophyll content in highly contaminated substrates (Alami et al., 2025). However, the current body of literature remains fragmented, with most work conducted using greenhouse conditions or pot trials. Comprehensive field evaluations, long-term monitoring, and assessments of ecological risks and microbial community dynamics, together with clear information regarding the involvement of ACC deaminase are still lacking.

## 4 Summary and conclusions

Earlier studies have shown that inhibition of the growth and development of many plants is often a consequence of environmental (either abiotic or biotic) stresses (Abeles et al., 1992). This plant growth inhibition results both from the direct effects of the environmental stress on the plant as well as the production of stress ethylene in those stressed plants. Some of these environmental stresses include temperature extremes, high light, flooding, drought, high salt, toxic metals, organic contaminants, radiation, wounding, insect predation, nematodes predation, as well as the negative effects of viruses, and pathogenic fungi and bacteria (Abeles et al., 1992). One way of lowering stress ethylene production in plants and thereby lowering the negative effects of environmental stresses is through the use of soil bacteria that contain the enzyme ACC deaminase (Glick et al., 2007). Given the fact that most rhizobial strains typically have only a low level of ACC deaminase, this enzyme level is often insufficient to rescue plants from the deleterious effects of environmental stresses. However, rhizobial strains with low levels of ACC deaminase can become much more effective at combatting the effects of environmental stress when the rhizobia strain is either part of a consortium that includes a free-living ACC deaminase-containing plant growth-promoting bacterium, or a NRE synthesizing the enzyme, or the rhizobial strain has been genetically transformed to express ACC deaminase activity from a free-living bacterium (Figure 1). Finally, it needs to be emphasized that notwithstanding the success in using free-living ACC deaminase-containing plant growth-promoting bacteria to overcome a wide range of environmental stresses, to date, genetically modified rhizobia or rhizobia in ACC deaminase-containing consortia have only been tested (albeit successfully) with a limited range of environmental stresses.
